# Supplemental Milestones for Emergency Medicine Residency Programs: A Validation Study

**DOI:** 10.5811/westjem.2016.10.31499

**Published:** 2016-11-15

**Authors:** Andrew R. Ketterer, David H. Salzman, Jeremy B. Branzetti, Michael A. Gisondi

**Affiliations:** * Northwestern University Feinberg School of Medicine, Department of Emergency Medicine, Chicago, Illinois; † Feinberg Academy of Medical Educators, Department of Medical Education, Chicago, Illinois; ‡ University of Washington School of Medicine, Division of Emergency Medicine, Seattle, Washington

## Abstract

**Introduction:**

Emergency medicine (EM) residency programs may be 36 or 48 months in length. The Residency Review Committee for EM requires that 48-month programs provide educational justification for the additional 12 months. We developed additional milestones that EM training programs might use to assess outcomes in domains that meet this accreditation requirement. This study aims to assess for content validity of these supplemental milestones using a similar methodology to that of the original EM Milestones validation study.

**Methods:**

A panel of EM program directors (PD) and content experts at two institutions identified domains of additional training not covered by the existing EM Milestones. This led to the development of six novel subcompetencies: “Operations and Administration,” “Critical Care,” “Leadership and Management,” “Research,” “Teaching and Learning,” and “Career Development.” Subject-matter experts at other 48-month EM residency programs refined the milestones for these subcompetencies. PDs of all 48-month EM programs were then asked to order the proposed milestones using the Dreyfus model of skill acquisition for each subcompetency. Data analysis mirrored that used in the original EM Milestones validation study, leading to the final version of our supplemental milestones.

**Results:**

Twenty of 33 subjects (58.8%) completed the study. No subcompetency or individual milestone met deletion criteria. Of the 97 proposed milestones, 67 (69.1%) required no further editing and remained at the same level as proposed by the study authors. Thirty milestones underwent level changes: 15 (15.5%) were moved one level up and 13 (13.4%) were moved one level down. One milestone (1.0%) in “Leadership and Management” was moved two levels up, and one milestone in “Operations and Administration” was moved two levels down. One milestone in “Research” was ranked by the survey respondents at one level higher than that proposed by the authors; however, this milestone was kept at its original level assignment.

**Conclusion:**

Six additional subcompetencies were generated and assessed for content validity using the same methodology as was used to validate the current EM Milestones. These optional milestones may serve as an additional set of assessment tools that will allow EM residency programs to report these additional educational outcomes using a familiar milestone rubric.

## INTRODUCTION

The Accreditation Council for Graduate Medical Education (ACGME) has fully implemented the Next Accreditation System, a framework of continuous accreditation that uses outcomes-based, specialty-specific milestones for resident assessment.^1^,^2^ The ACGME, the Residency Review Committee for Emergency Medicine (RRC-EM), and the American Board of Emergency Medicine (ABEM) co-convened the Emergency Medicine (EM) Milestones Working Group to create the EM Milestones.^3^,^4^ As described by ABEM, “the EM Milestones are a matrix of the knowledge, skills, abilities, attitudes, and experiences that should be acquired during specialty training in EM.”^5^ Validated and published in 2013, the EM Milestones are used to track and report residents’ progress in 23 different content domains described as subcompetencies.^3^,^4^,^6^

Residency programs in EM are configured in 36-month or 48-month formats. The EM Milestones are used by all ACGME-accredited EM residency programs, regardless of program length.^1^ However, EM residency programs with a 48-month training format are expected to provide the RRC-EM with “justification describing the additional educational goals and outcomes to be achieved by residents in the incremental 12 months of education.”^1^ Different programs use this additional training time in different ways, including extra elective time, built-in mini-fellowships, scholarly tracks, and other means of academic and professional development.^7^ The EM Milestones were not designed to reflect the “additional educational goals and outcomes” of 48-month residency programs, but rather were intended to evaluate resident progress during training irrespective of program length.^1^

The authors of this study developed supplemental milestones to track their residents’ progress within domains not reflected in the current EM Milestones (Appendix). Importantly, these supplemental milestones were designed to augment the self-study process by providing a concrete means of resident assessment using the already-familiar EM Milestone format.^8^

The goals of this study were to assess the content validity of these supplemental milestones, and to refine them using the same methodology established by the EM Milestone Working Group to create the current EM Milestones.^3^,^4^

## METHODS

### Study Design, Setting and Population

This was a cross-sectional survey of program directors (PD) at ACGME-accredited, allopathic, 48-month EM residency programs during the study period. Associate/assistant program directors (APD) were excluded from this survey. This study was considered exempt by the institutional review board of Northwestern University.

### Supplemental Milestone Development

A seven-person panel of EM educators at two 48-month training programs, including an active PD and multiple APDs, convened to create supplemental milestones that described educational domains common to many, but not all, 48-month training programs, that are not otherwise reflected in the EM Milestones. Using an iterative process, six new subcompetencies were drafted by consensus, each with its own set of defining milestones, which were sequentially reviewed and refined by the authors. Next, four subject-matter experts consisting of experienced APDs at other 48-month EM training programs were tasked with reviewing content, survey format, quality and clarity of instructions, and usability of these supplemental milestones. Their comments were incorporated into the final version of our proposed supplemental milestones. Our subject-matter experts were asked to keep the content of this study confidential from the intended study subjects (i.e. their respective PDs).

### Survey Administration and Content

The validation phase used a computer-based survey platform powered by Qualitrics^©^ LLC (Provo, Utah). We emailed the survey to all eligible subjects between February 6, 2015, and May 31, 2016, during which a total of five interval reminders were sent to nonresponders. We de-identified all data, and individuals’ responses were kept confidential from the study authors.

For each of the individual six subcompetencies proposed, respondents reviewed a complete list of corresponding milestones, presented in randomized order. Respondents were asked to click and drag each milestone to an area on their screen corresponding to one of five levels. Like the original EM Milestones project,^4^ we used the Dreyfus model of skill acquisition^9^ to define levels of competency from novice (Level 1) to expert (Level 5, indicative of aspirational performance). Detailed instructions for this task were included in the survey instrument, providing a functional description of the Dreyfus model to survey respondents. The option to mark individual milestones as inappropriate for inclusion was also provided as part of the survey, as was a free-text area for comments. The primary outcome of this study was the frequency of milestone assignment into a specific level designation.

The authors then used the survey results to amend the inclusion or assignment of milestones within a level using a set of predefined decision rules described in the validation study of the current EM Milestones.^4^ The decision rules included the following:

- Milestones were not altered if 50% or more of respondents assigned a milestone to the same level as was proposed by our study team.
- Milestones were deleted if more than 50% of respondents recommended deletion.
- The assignment of a milestone level was changed when 50% or more of respondents assigned a milestone to a different level than was proposed by our study team.
- If a milestone was not assigned to a single level by more than 50% of respondents, the milestone was assigned to the level at which a cumulative 50% of respondents chose that level or below.

### Data Analysis

We tallied response rates for each of the milestones using the Qualtrics^©^ survey software and entered their allocations into an Excel (version 15.14, Microsoft^©^) spreadsheet. Frequencies and cumulative frequencies were calculated and charted, decision rules applied, and final milestone levels assigned.

## RESULTS

Of the 34 eligible subjects, one was excluded because of his authorship on this paper and involvement in developing the proposed supplemental milestones. Twenty of the remaining 33 recipients (58.8%) completed the survey within the study period. Responses were received from five of six Society for Academic Emergency Medicine (SAEM) geographic regions (Table 1).^10^

Of the 97 proposed supplemental milestones, 67 (69.1%) were kept at the same level as proposed by the study authors, without further editing. Three of the proposed subcompetencies demonstrated high rates of agreement between the survey respondents and the proposed milestones: Eleven of 15 milestones (73.3%) in “Operations and Administration” were kept at the same level as proposed by the study authors (s 1 and 2); for “Critical Care,” 11 of 14 milestones (78.6%) were unchanged, and for “Leadership and Management” 13 of 16 milestones (81.3%) were unchanged. The remaining three subcompetencies showed moderate levels of agreement: For “Research,” 9 of 17 milestones (52.9%) were kept at the same level as proposed by the authors; for “Teaching and Learning,” 12 of 18 milestones (66.7%) were unchanged, and for “Career Development,” 11 of 17 milestones (64.7%) were unchanged.

In all, 30 milestones underwent level changes based on survey responses; 15 (15.5%) were moved one level up and 13 (13.4%) were moved one level down. One milestone (1.0%) in “Leadership and Management” was moved two levels up, and one milestone in “Operations and Administration” was moved two levels down. One milestone in “Research” met decision rules criteria to be moved one level up; however, this milestone was ultimately kept at its original level after review by the study authors (Table 2).

No milestones met criteria for deletion. The final distribution of milestones for the six supplemental subcompetencies are: 9 in Level 1 (9.3% of 97 total milestones), 25 in Level 2 (25.8%), 26 in Level 3 (26.8%), 22 in Level 4 (22.7%), and 15 in Level 5 (15.5%).

## DISCUSSION

This is the first study to replicate the methodology used by the EM Milestones Working Group to create a set of supplemental milestones.^3^,^4^ In our study, the content validity of these milestones was assessed specifically for potential use within a cohort of 48-month EM residency programs. Similar to the development of the EM Milestones, this study shows that a set of “objective, observable actions” can be assigned by PDs “into progressive levels of competency acquisition” for the assessment of residents in distinct educational domains.^4^ The ACGME Program Requirements for EM, as recently updated by the RRC-EM, mandate that 48-month EM programs provide an educational justification for the additional training time in their programs.^1^ The existing EM Milestones were meant to capture clinical competency for all EM residents, and therefore they may not reflect added educational goals and objectives for the additional 12 months of training in 48-month residency programs. The subcompetencies developed in this study reflect six potential content domains that could be used to meet the aforementioned educational justification required of 48-month EM programs. We may now assess residents’ skill acquisition in these domains as a progression to competence, using a reporting framework similar to the standard EM Milestones.^8^,^11^

These supplemental milestones are not meant to replace, direct, or alter the existing curricula of any of the other 48-month EM residency programs. Each 48-month program may use their additional training time to meet their own unique program-specific aims.^7^,^8^,^11^ The supplemental milestones described in this study were developed with the intention that they might serve as a potential tool to assess and track already-existing curricula. We chose subcompetency domains thought to be common to 48-month EM programs, thereby performing this validation study within our cohort of programs. We recognize that topics such as “Critical Care” might have appeal to most but not all 48-month EM programs, while topics such as “Research” might be common to many but not the majority of these programs. Certainly there are other domains that could be explored using similar methodology to this study, for example, the validation of milestones in areas such as global health, emergency medical services, or ultrasound. Obviously not all programs aim to train residents in these additional domains, but for those that do, the option to assess and report residents’ skill acquisition in these content areas may be appealing. Residency programs may choose to use this methodology to create similar self-study assessment tools to track their residents’ progress within specific elements of their current curricula. An alternative approach would be to use a similarly robust development process to generate these tools, and forego the content validation phase by external experts. This is particularly appealing in light of the labor-intensive nature of external content validation, as well as the relatively low frequency of level reassignment by survey respondents. However, we felt that the high stakes of assessment imparted by the milestones suggests a need for the robust content validation process described in this study, and we would recommend a similar approach to the development of such tools in the future.

The ACGME Next Accreditation System includes a sequence of eight steps intended to guide programs in conducting a self-study. Their recommended fourth step is to “Aggregate and analyze data to generate a longitudinal assessment of the program’s improvement.”^8^ In addition to tracking individual resident progress, our proposed supplemental milestones may be used to assess the impact of longitudinal curricular changes. The impact of changes in program curricula may be monitored by tracking cumulative resident progress as class cohorts using these supplemental milestones year after year. We believe this satisfies the ACGME’s recommended fourth step of self-study by generating data that track how well a program’s own specific educational goals are being met by its trainees, and how this progress changes in response to curriculum modification over time. The data generated by these tools could also be used to strengthen a program’s presentation for internal review or ACGME site visit.

With the exception of one milestone, our final set of supplemental milestones reflects the positions assigned according to the aforementioned set of predefined decision rules. The single exception was milestone 2.6 in the “Research” subcompetency (“Identifies and explains methods of statistical analysis commonly used in the medical literature”). The survey respondents assigned that milestone to level 3 by a slight majority; however, we felt it represented a stepwise progression in sophistication of research skills between “describing common research designs” (milestone 1.1) and “leading critical discussions of medical literature” (milestone 3.2). Finally, we felt justified in leaving this milestone at its originally assigned level because only a very slight majority of respondents indicated that it should be assigned to level 3; if a single respondent had assigned it to level 2 rather than level 3, the majority would have agreed with the study authors.

## LIMITATIONS

Our study includes some important limitations. Our overall response rate represents just over half of the potential subjects, which could indicate results that are biased and not representative of our intended population. However, multiple factors may mitigate this potential bias. Our response rate of 58.8% is higher than that in the original EM Milestone validation study, which had a response rate of 36.6%.^4^ While the original study sampled a larger population of “key faculty,”^4^ we focused specifically on PDs in this study. Therefore, while there may be fewer overall respondents, the potential for higher quality respondent data from directors who are experienced with the milestone process may be less prone to bias than the more general population of key faculty surveyed by Korte et al.^4^ Moreover, the survey respondents had a broad geographical distribution, suggesting that the final version of the supplemental milestones accurately represents the attitudes of PDs at a variety of EM training programs. This protects against potential bias from the attitudes of any one geographic region, although this protection is limited by the response of only a minority of programs in New England, and none of the eligible programs in the Great Plains region. Finally, the methodology of our study included multiple layers of data acquisition and review beyond simple collection of survey responses. This includes our initial solicitation of expert opinions to generate the new milestone domains and content, subject-matter experts to review of the drafted milestones, survey respondents’ assignments of milestones to specific levels, and final study group review of all generated data to ensure that each milestone was an appropriate match for the level to which our validation cohort assigned.

The original proposals for the subcompetencies and milestones in this study were all written and edited by faculty from two institutions. While each faculty member had content expertise pertinent to their tasked subcompetency (APD, PD, operations directors, etc.), the content selected for inclusion may be biased by specific institutional strengths, norms, or expectations. We believe the use of external subject matter experts for review of the proposed milestones, as well as the use of directors of 48-month EM programs as subjects, mitigates this potential bias.

As this validation study was conducted among programs of a similar length of training, we chose to title this manuscript, “Supplemental milestones for EM residency programs.” It is likely that these tools could also be used by 36-month EM programs that have program-specific aims and curricula that are similar to those in our validation cohort, much like the current EM Milestones themselves. Similarly, there may be other potential educational domains that are more relevant to specific programs than the six options resulting from this study. Programs may choose to adopt one or more of the subcompetencies that we developed, or instead create ones that are more ideally suited to their needs. This study demonstrates a process that can be followed by any cohort of similar residency programs.

As with the current EM Milestones, no editing or review of our proposed milestones based on real-world implementation has been performed. It is conceivable that such post-hoc experiential data may prove valuable enough to necessitate editing of the milestone content, phrasing, or level assignment, as is planned in future iterations of the Milestones by the ACGME and RRC-EM.^12^,^13^

## CONCLUSION

This study resulted in the development of six supplemental subcompetencies and corresponding milestones for EM that were assessed for content validity among a cohort of 48-month EM residency program directors, using the methodology of the EM Milestones Working Group. These optional tools may be used to track residents’ skill acquisition in educational domains that are distinct from those of the original EM Milestones. Further study will be needed to assess the implementation and longitudinal utility of these new milestones by residency programs in EM.

## 

## Figures and Tables

**Figure 1:**
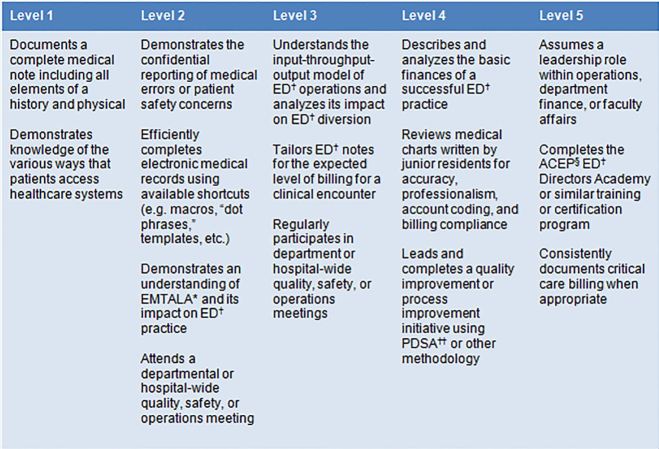
Original matrix of the supplemental milestone “Operations and Administration” as proposed by the study authors. ^*^Emergency Medical Treatment and Labor Act; †Emergency Department; ††Plan-Do-Study-Act; §American College of Emergency Physicians.

**Figure 2:**
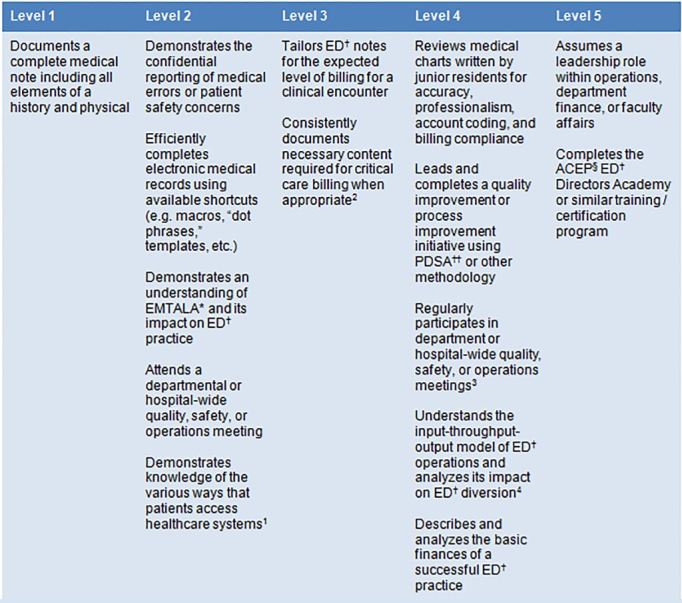
Revised matrix for the proposed supplemental milestone, “Operations and Administration, “based on survey results. 1. This milestone was originally at level 1 and was moved up 1 level. 2. This milestone was originally at level 5 and was moved down 2 levels. 3. This milestone was originally at level 3 and was moved up 1 level. 4. This milestone was originally at level 4 and was moved up 1 level. ^*^Emergency Medical Treatment and Labor Act; †Emergency Department; ††Plan-Do-Study-Act; §American College of Emergency Physicians.

**Table 1:** Geographic data of emergency medicine program directors of 48-month programs who responded to a survey regarding proposed new milestones created to supplement existing EM milestones.

Society for Academic Emergency Medicine Region	Number of Respondents	Number of 48-month Programs in Region	% Total
New England	5	13	38.5
Mid-Atlantic	4	4	100
Southeastern	1	1	100
Midwest	2	2	100
Great Plains	0	3	0
Western	8	11	72.7

**Table 2:** Frequency of supplemental milestone-level changes based on survey results.

Subcompetency	No. milestones	Single-level change (% total)	Two-level change (% total)	Deleted milestones
Operations and Administration	15	3 (20.0%)	1 (6.7%)	0
Research	17	8 (47.1%)	0 (0.0%)	0
Critical Care	14	3 (21.4%)	0 (0.0%)	0
Teaching and Learning	18	6 (33.3%)	0 (0.0%)	0
Career Development	17	6 (35.3%)	0 (0.0%)	0
Leadership and Management	16	2 (12.5%)	1 (6.3%)	0
