# Supplementary material for: Supplemental Milestones for Emergency Medicine Residency Programs: A Validation Study
Source: West J Emerg Med. 2016 Nov 15;18(1):69–75. doi: 10.5811/westjem.2016.10.31499 (PMC5226766; doi:10.5811/westjem.2016.10.31499)
Supplement: Supplementary file 1 [file wjem-18-69-s001.pdf]

Supplemental EM Milestones: Operations and Administration					
Has not achieved Level 1	Level 1	Level 2	Level 3	Level 4	Level 5
	Documents a complete medical note including all elements of a history and physical<br><br>Demonstrates knowledge of the various ways that patients access healthcare systems	Demonstrates the confidential reporting of medical errors or patient safety concerns<br><br>Efficiently completes electronic medical records using available shortcuts (e.g. macros, “dot phrases,” templates, etc.)<br><br>Demonstrates an understanding of EMTALA and its impact on ED practice<br><br>Attends a departmental or hospital-wide quality, safety, or operations meeting	Tailors ED notes for the expected level of billing for a clinical encounter<br><br>Consistently documents necessary content required for critical care billing when appropriate	Reviews medical charts written by junior residents for accuracy, professionalism, account coding, and billing compliance<br><br>Leads and completes a quality improvement or process improvement initiative using PDSA or other methodology<br><br>Regularly participates in department or hospital-wide quality, safety, or operations meetings<br><br>Understands the input-throughput-output model of ED operations and analyzes its impact on ED diversion	Assumes a leadership role within operations, department finance, or faculty affairs<br><br>Completes the ACEP ED Directors Academy or similar training / certification program<br><br>Describes and analyzes the basic finances of a successful ED practice
☐	☐ ☐	☐ ☐	☐ ☐	☐ ☐	☐
Comments:					

Supplemental EM Milestones: Research					
Has not achieved Level 1	Level 1	Level 2	Level 3	Level 4	Level 5
	Describes common research study designs (e.g. RCT, case-control, cohort, etc.)	Identifies the study design and research methods of individual journal articles<br><br>Demonstrates the ability to properly phrase a research question<br><br>Participates in critical discussions of medical literature<br><br>Understands the scope and limitations of peer-reviewed medical journals<br><br>Recognizes common methodological biases that limit the practical application of a given journal article<br><br>Identifies and explains methods of statistical analysis commonly used in medical literature (e.g. chi-square test, p-values, 95% confidence intervals, etc.)	Critically analyzes publications that are not peer-reviewed, such podcasts and specialty organization newsletters<br><br>Leads critical discussions of medical literature<br><br>Obtains IRB or human subject committee approval for a proposed research project	Authors a research manuscript of publishable quality<br><br>Writes and submits a grant for funding of a scholarly project<br><br>Presents findings from own research or a scholarly project at a peer gathering<br><br>Publishes a research manuscript in peer-reviewed medical literature	Obtains grant funding for a research project<br><br>Completes ACEP EMBRS course, or equivalent certification<br><br>Completes a research fellowship or advanced degree in a related field
☐	☐	☐ ☐	☐ ☐	☐ ☐	☐ ☐
Comments:					

Supplemental EM Milestones: Critical Care

Has not achieved Level 1	Level 1	Level 2	Level 3	Level 4	Level 5
	Seeks appropriate supervision when caring for a critically ill patient<br><br>Completes requisite critical care certification(s): ACLS, PALS, NRP, and/or ATLS certification<br><br>Effectively communicates a treatment plan to next-of-kin or surrogate decision makers	Reliably assesses ABCs and primary survey for a critically ill or injured patient<br><br>Understands the pathophysiology and management of the major categories of shock<br><br>Knows the indications for the common means of invasive monitoring	Correctly interprets diagnostic data in the critically ill patient and tailors specific therapeutic interventions accordingly (eg, ABG for vent management, CVP for resuscitation, etc)<br><br>Effectively leads a resuscitation or cardiac arrest<br><br>Compassionately communicates death notifications to family members<br><br>Synthesizes data from all forms of input (history, exam, diagnostics, etc.) to create a complete care plan for critically ill patients, regardless of age, comorbidity, or other challenging factors	Coordinates multidisciplinary team and family meetings in planning the ongoing care for a critically-ill patient<br><br>Knows indications for rare or complex resuscitative interventions (such as ECMO, IABP, cardiac bypass or ED thoracotomy)	Completes a critical care fellowship<br><br>Obtains board certification in critical care
☐	☐ ☐	☐ ☐	☐ ☐	☐ ☐	☐

Comments:

Supplemental EM Milestones: Teaching and Learning					
Has not achieved Level 1	Level 1	Level 2	Level 3	Level 4	Level 5
	Accepts feedback from teachers in a professional manner	Uses basic teaching approaches in the clinical environment<br><br>Demonstrates the ability to incorporate lessons learned from prior patient experiences or clinical questions<br><br>Provides feedback to teachers in an objective, professional manner<br><br>Identifies questions or issues from clinical encounters to drive post-clinical learning and/or self-study	Assists with sessions run by senior instructors or teaches previously developed content (e.g. small group simulation, interest group sessions) to junior learners<br><br>Regularly incorporates teaching of others during each patient encounter<br><br>Reliably highlights a “teachable moment” for members of the care team<br><br>Teaches large or small groups in traditional settings (e.g. lecture, discussion groups)<br><br>Consistently employs debriefing and feedback techniques with learners<br><br>Regularly seeks and integrates feedback to improve teaching methods	Utilizes advanced teaching strategies (e.g. integration of technology, active learning strategies, etc.)<br><br>Effectively balances clinical teaching with patient care responsibilities, regardless of clinical volume<br><br>Effectively teaches other providers how to teach	Designs and coordinates the implementation of novel curricula or educational initiatives<br><br>Completes ACEP Teaching Fellowship, or equivalent<br><br>Completes education fellowship or advanced degree in education<br><br>Obtains a formal leadership role in medical education (e.g. residency or medical student program leader, PD, APD, Assoc Dean, etc)
☐	☐	☐ ☐	☐ ☐	☐ ☐	☐ ☐
Comments:					

Supplemental EM Milestones: Career Development					
Has not achieved Level 1	Level 1	Level 2	Level 3	Level 4	Level 5
	Identifies the purpose of an advisor or mentor	Seeks advice from faculty members or senior staff on potential scholarly projects, career options, or career development opportunities<br><br>Broadly identifies several potential areas of career focus for emergency physicians<br><br>Identifies possible mentors at home institution for personal career interests<br><br>Identifies potential sources of information for investigating career options (websites, mentors, etc.)<br><br>Collaborates with colleagues who share similar scholarly or professional interests	Describes realistic 5- and/or 10-year career plans in the context of personal longitudinal career focus<br><br>Demonstrates the ability to balance short-term residency demands with long-term career development planning<br><br>Engages in a sustained mentor-mentee and/or advising relationship<br><br>Explores a potential professional niche in healthcare through focused study or experiential learning	Identifies and pursues an ideal practice environment for the attainment of personal goals<br><br>Obtains post-graduate employment that allows for continued development in chosen professional niche<br><br>Engages as a mentor for attainment of a junior physician's career goals<br><br>Continually acquires and refines requisite skills for chosen area of focus	Attains a regional and/or national leadership role related to a chosen professional niche<br><br>Attains national and/or international recognition for contributions to a specific area of focus within medicine<br><br>Consistently participates in regional and/or national groups within a focused area of emergency medicine
☐	☐	☐ ☐	☐ ☐	☐ ☐	☐ ☐ ☐
Comments:					

Supplemental EM Milestones: Leadership & Management					
Has not achieved Level 1	Level 1	Level 2	Level 3	Level 4	Level 5
	Identifies immediate supervisor(s) when working clinically in the ED<br><br>Identifies chief residents and their role in the residency<br><br>Describes residency leadership structure	Participates in any required committee work<br><br>Joins organizations of personal or professional importance<br><br>Demonstrates the ability to describe the organizational chart of own emergency department and the role of all department leaders<br><br>Demonstrates the ability to describe the organizational chart of local hospital or health system	Actively serves on a local committee as the department resident representative (or other equivalent leadership role)<br><br>Actively participates in organizations of personal or professional importance<br><br>Consistently performs critical self-assessment of leadership skills and routinely seeks ongoing developmental opportunities<br><br>Demonstrates ongoing improvement of professional skills through voluntary or extracurricular training courses or experiential learning opportunities<br><br>Correctly involves organizational resources within the hospital to clinical practice (calls risk management, initiates a disaster response, notifies ED medical director, etc.)	Leads a committee, task force, or organization of any size<br><br>Identifies leadership opportunities for senior trainees	Effectively manages the educational and clinical growth of junior physician leaders and/or other hospital staff<br><br>Attains advanced leadership positions in one or more healthcare organizations (e.g. local medical staff, APT committee, BOD of a professional committee)
☐	☐ ☐	☐	☐ ☐ ☐	☐ ☐	☐
Comments:					
